# Small Intestinal Nematode Infection of Mice Is Associated with Increased Enterobacterial Loads alongside the Intestinal Tract

**DOI:** 10.1371/journal.pone.0074026

**Published:** 2013-09-10

**Authors:** Sebastian Rausch, Josephin Held, André Fischer, Markus M. Heimesaat, Anja A. Kühl, Stefan Bereswill, Susanne Hartmann

**Affiliations:** 1 Institute of Immunology, Department of Veterinary Medicine, Freie Universität, Berlin, Germany; 2 Department of Neuropathology, Charité - University Medicine Berlin, Berlin, Germany; 3 Department of Microbiology and Hygiene, Charité - University Medicine Berlin, Berlin, Germany; 4 Department of Internal Medicine, Rheumatology and Clinical Immunology/Research Center ImmunoSciences (RCIS), Charité - University Medicine Berlin, Berlin, Germany; Virginia Tech University, United States of America

## Abstract

Parasitic nematodes are potent modulators of immune reactivity in mice and men. Intestinal nematodes live in close contact with commensal gut bacteria, provoke biased Th2 immune responses upon infection, and subsequently lead to changes in gut physiology. We hypothesized that murine nematode infection is associated with distinct changes of the intestinal bacterial microbiota composition. We here studied intestinal inflammatory and immune responses in mice following infection with the hookworm *Heligmosomoidespolygyrus* *bakeri* and applied cultural and molecular techniques to quantitatively assess intestinal microbiota changes in the ileum, cecum and colon. At day 14 post nematode infection, mice harbored significantly higher numbers of γ*-Proteobacteria/Enterobacteriaceae* and members of the *Bacteroides* */Prevotella* group in their cecum as compared to uninfected controls. Abundance of Gram-positive species such as *Lactobacilli*, *Clostridia* as well as the total bacterial load was not affected by worm infection. The altered microbiota composition was independent of the IL-4/-13 – STAT6 signaling axis, as infected IL-4Rα^-/-^ mice showed a similar increase in enterobacterial loads. In conclusion, infection with an enteric nematode is accompanied by distinct intestinal microbiota changes towards higher abundance of gram-negative commensal species at the small intestinal site of infection (and inflammation), but also in the parasite-free large intestinal tract. Further studies should unravel the impact of nematode-induced microbiota changes in inflammatory bowel disease to allow for a better understanding of how theses parasites interfere with intestinal inflammation and bacterial communities in men.

## Introduction

Nematodes are the most prevalent worms worldwide dwelling the intestine of humans. Infections with *Ascaris* *lumbricoides* , *Trichuris* *trichura* , *Ancylostoma* *duodenale* and *Necator americanus* generally lead to persistent intestinal colonization [1]. Our study aimed to analyse the influence of infections with the murine hookworm model *Heligmosomoidespolygyrus* *bakeri* ( *H* . *p* *. bakeri* ) [2] on the composition of the bacterial microbiota in distinct compartments of the murine intestinal tract. *H* . *p* *. bakeri* inhabits an environment colonized by a large number of commensal and symbiotic microorganisms. The commensal host microbiota is essential for physiological processes, such as nutrient acquisition, maintenance of epithelial barrier function, and mucosal immune homeostasis [3,4]. Information regarding potential quantitative and qualitative microbiota changes induced by intestinal worm infections are scarce, but of major interest, given that distinct commensal species have been shown to be involved in development and perpetuation of inflammatory bowel disease (IBD) in humans [5,6]. In addition, parasitic nematodes are considered as a therapeutic option for the treatment of IBD [7] given that trials applying helminth eggs to patients with Crohn’s disease and ulcerative colitis resulted in disease remission [8]. Suppression of intestinal inflammation and induction of regulatory immune responses by parasitic nematode infections have been demonstrated in various IBD models (reviewed in 9). Furthermore, enteropathogenic infections and murine experimental colitis lead to dramatic changes in the intestinal ecosystem with alterations in the microbiota composition [10]. Although these models reflect a multifactorial etiology of intestinal inflammation, the resulting changes in the composition of the intestinal microbial community are consistently seen as a preferential overgrowth of the Gram-negative *Enterobacteriaceae*. Hence, it is under debate whether the phenomenon of “dysbiosis” in animal models and human IBD is primarily resulting from intestinal inflammation or initiating and perpetuating the inflammatory condition per se (“hen and egg”) [11–13].

In the presented study, we investigated the alterations of the intestinal bacterial microbiota composition following a gastrointestinal hookworm infection applying cultural and PCR-based techniques. We here show that i) *H* . *p* *. bakeri* infection was accompanied by a shift towards increased abundance of Gram-negative commensal species. ii) Remarkably, the most distinct changes of the microflora were determined distal from the site of acute nematode infection, i.e. in the cecum and colon. iii) IL-4Rα^-/-^ mice showed similar changes in the microbiota composition after nematode infection, revealing that changes induced by the IL-4/-13 – STAT-6 signalling axis in the intestines are not involved in permitting the outgrowth of Gram-negative bacteria.

## Materials and Methods

### Ethics Statement

All animal experiments were performed in strict accordance with the national animal protection guidelines and approved by the appropriate ethics committee (Landesamt für Gesundheit und Soziales, Berlin, Germany; Permit Number G0363/10).

### Mice, parasites and infections

Female C57BL/6 mice (purchased from Charles River) and IL-4Rα^-/-^ mice (C57BL/6 background, a kind gift from M. Löhning, DRFZ, Berlin) were kept under specific pathogen-free conditions (light/dark cycles of 12h) in ventilated cabinets in cages equipped with standard bedding, filter tops, environmental enrichment and fed standard diet and autoclaved water ad libitum. 200 *H* . *p* *. bakeri* infective larvae (L3) were applied in 100µl water orally to 6-8 week old mice. Control mice received water in which the L3 had been stored at 4°C. Mice were sacrificed by cervical dislocation after inhalational (isoflurane) anesthesia on day 6, 14 or 28 after *H* . *p* *. bakeri* infection.

### Cell culture, gut homogenates and cytokine detection

Mesenteric lymph node cells (mLNC) were isolated aseptically and single cell suspensions were plated in RPMI 1640 containing 5% fetal calf serum (both from BioChrom, Germany), 20 mM L-glutamine, 100 U/ml penicillin and 100 µg/ml streptomycin (PAA, Germany) as triplicates on 96-well plates. Cultures were kept at 37°C and 5% CO_2_ with 3.5×10^5^ cells in a total volume of 200 µl. Stimulation with concanavalin A (ConA, 2 µg/ml) was performed for 48 h, cultures with adult worm antigen extracts (40 µg/ml) were kept for 72 h. Supernatants were harvested and stored at -80°C. Culture supernatants were assayed by ELISA (kits from BD Biosciences and R&D Systems) for IL-4, IL-13, IL-10, IL-12p70, IL-17A, IFN-γ, and TGF-β1 according to the manufacturer’s instructions.

### Histopathology

Intestinal tissue samples were fixed in 3.7% phosphate-buffered formalin, embedded in paraffin and used for cross sections. The histopathological score in response to infections was determined using hematoxylin/eosin stained cross sections. The grade of inflammation of small intestine, cecum and colon was evaluated according to the following parameters: (1) epithelial damage, (2) edema development, (3) inflammatory cell infiltration, (4) crypt depth/villus length. Goblet cells were quantified in periodic acid and Schiff’s reagent stained sections (10 high power fields/section).

### Cultural analysis of the intestinal microflora

Quantitative cultural analyses were performed as described earlier [14]. In brief, ileal, cecal and colonic luminal contents were immediately resuspended in sterile PBS. Serial dilutions were plated onto respective solid media, bacteria were grown at 37 °C for two days under aerobic and obligate anaerobic conditions and bacterial loads expressed as colony forming units (CFU) per gram luminal content (wet weight).

### Molecular analysis of the intestinal microbiota

DNA extractions from intestinal luminal contents were prepared as described previously [14]. In brief, DNA extracts and plasmids were quantified by using Quant-iT PicoGreen reagent (Invitrogen, UK) and all adjusted to 1 ng DNA/µl.

The abundance of specific intestinal bacterial groups was measured by qPCR with group-specific 16S rRNA gene primers (Tib MolBiol, Germany) as decribed previously [15,16]. As reference for quantification standard curves with tenfold serial dilutions of plasmids (ranging from 2×10^8^ to 2×10^2^ copies) were generated for each run. The real-time PCR primers were first used to amplify cloned 16S rDNA of reference strains (see Table 1). The number of 16S rRNA gene copies / ng DNA of each sample was determined. Frequencies of the given bacterial groups were calculated proportionally to the eubacterial (V3) amplicon.

**Table 1:** 16S rRNA gene group-specific primer for quantitative Real Time-PCR^**a**^.

**Target**	**Reference Strain**	**Primer sequence (5´ to 3´) and Orientation^b^**	**Reference**
Domain Bacteria (targets 16S V3 region)	*Escherichia coli* ATCC 25922	F: CGGYCCAGACTCCTACGGG, R:TTACCGCGGCTGCTGGCAC	[50]
*Clostridium* *leptum* subgroup^c^	*Clostridium* *leptum* DSMZ 753	F: TTACTGGGTGTAAAGGG, R: TAGAGTGCTCTTGCGTA	[51]
*Clostridium* *coccoides* - *Eubacterium* *rectale* subgroup^d^	*Clostridium* *coccoides* DSMZ 935	F: AAATGACGGTACCTGACTAA, R: CTTTGAGTTTCATTCTTGCGAA	[52]
*Bacteroides* group^e^	*Bacteroides* *ovatus* DSMZ 1896	F: GAAGGTCCCCCACATTG, R: CAATCGGAGTTCTTCGTG	[53]
γ-Proteobacteria/ *Enterobacteriaceae*	*Escherichia coli* ATCC 25922	F: AAACTCAAATGAATTGACGG, R: CTTTTGCAACCCACTCC	[54]
*Lactobacillus* group^f^	*Lactobacillus acidophilus* DSM 20079	F: CACCGCTACACATGGAG, R: AGCAGTAGGGAATCTTCCA	[55,56]
*Bifidobacterium* genus	*Bifidobacterium* sp. (murine origin)	F: CTCCTGGAAACGGGTGG, R: GGTGTTCTTCCCGATATCTACA	[52,55,56]
*Enterococcus* genus	*Enterococcus faecalis* DSM 20478	F: CCTTATTGTTAGTTGCCATCATT, R: ACTCGTTGTACTTCCCATTGT	[57]
Mouse Intestinal *Bacteroides*	MIB plasmid 16-1	F: CCAGCAGCCGCGGTAATA, R: CGCATTCCGCATACTTCTC	[58]

a Modified from: Ref. [59]

b F, Forward R, Reverse

c including *Faecalibacterium* (Fusobacterium) *prausnutzii* *Clostridium* 16S rRNA cluster IV

d *Clostridium* 16S rRNA cluster XIVa/b

e including *Prevotella* and *Porphyromonas*

f including *Leuconostoc* , *Pediococcus* , *Aerococcus* and *Weissella* but not *Enterococcus* or *Streptococcus*

Genetic fingerprints were generated by PCR-based denaturing gradient gel electrophoresis (PCR-DGGE) as described previously [14].

### Statistical analysis

All experiments were performed with 4 to 6 mice per group and representative data are shown for at least two independent experiments as mean + SEM unless stated otherwise. Statistical analysis was performed using GraphPad Prism software using the two-tailed Mann–Whitney U-test. Values of p<0.05 were considered to be statistical significant.

## Results

### Histopathological mucosal changes following *H* . *p* *. bakeri* infection

**Figure 1:**
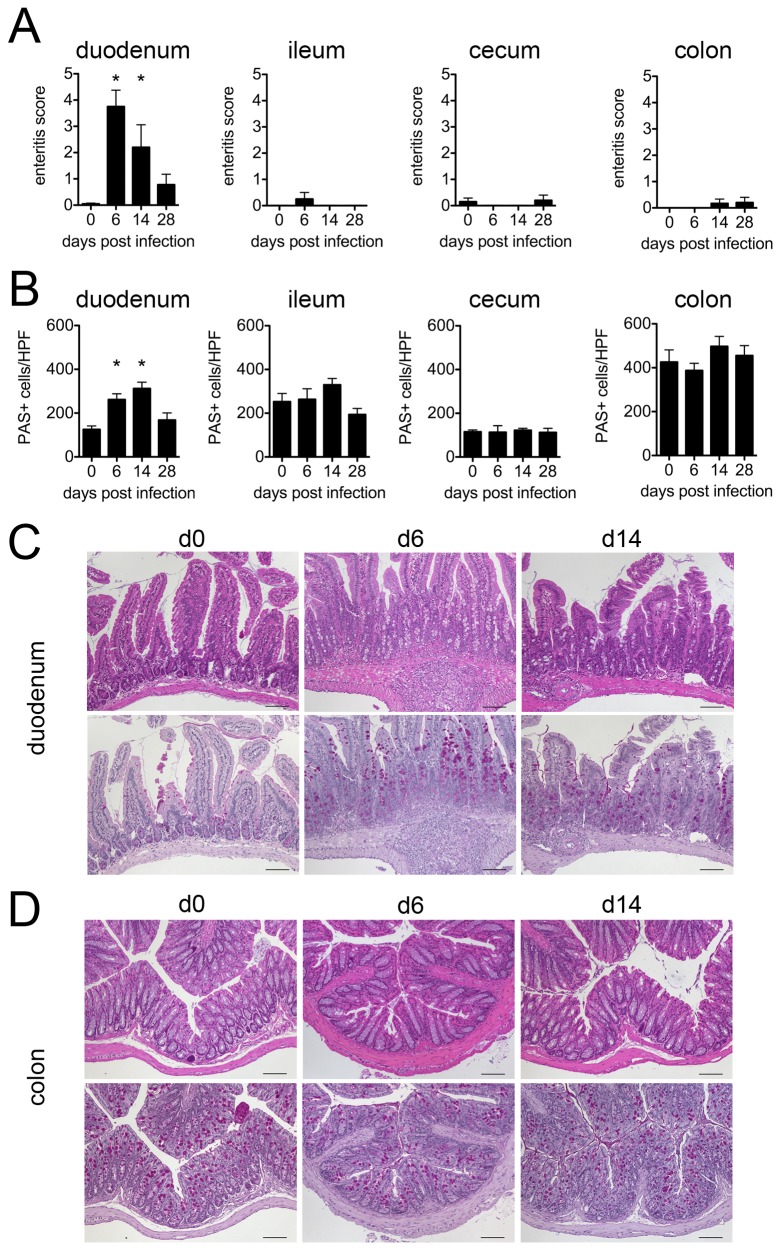
*H* . *p* *. bakeri* -induced intestinal immunopathology. (A) Enteritis development was evaluated at day 6, 14 and 28 post infection and naïve controls according to a scoring system based on epithelial damage, edema development, villus length, crypt depth and the size of inflammatory cell infiltrates. (B) Goblet cells counts as detected in intestinal cross sections by periodic acid/Schiff stain. Mean + SEM is shown for 5 mice per group; * p < 0.05. Representative cross sections from duodenum (C) and colon (D) stained with H&E (upper row) and periodic acid/Schiff (lower row, goblet cells marked by intense purple stain) for histopathological scoring and goblet cell quantification.

Infections with *H* . *p* *. bakeri* are generally well tolerated by mice and do not lead to signs of morbidity such as weight loss [17]. As the intestinal flora is perceptive to inflammatory changes of the gut [10,12,14], we surveyed the proximal small intestine as the site of nematode infection and, additionally, the worm-free large intestine histopathologically for signs of inflammation. Inflammatory scores based on morphological changes and the size of inflammatory cell infiltrates were compiled with cross sections from the duodenum, ileum, cecum and colon on day 6 (larval development), 14 (acute adult stage) and 28 post infection (chronic adult stage). When compared to naive controls, mice infected with *H* . *p* *. bakeri* for 6 and 14 days displayed a rather mild, but significantly elevated inflammatory score of the duodenum, but not ileum, cecum and colon (Figure 1 A, C, D). The locally restricted enteritis of the small intestine peaked during the larval and acute adult stage infection and declined thereafter during chronicity. In addition, development of enteritis was accompanied by local duodenal goblet cell hyperplasia while goblet cells counts remained unaltered in distal parts of the intestines (Figure 1 B, C, D).

Thus, infection with *H* . *p* *. bakeri* led to the development of a mild enteritis and increase of mucus producing goblet cells in the proximal small intestine, whereas distal parts of the intestinal tract appeared morphologically unaltered.

### Cytokine response on the peak of pathology

**Figure 2:**
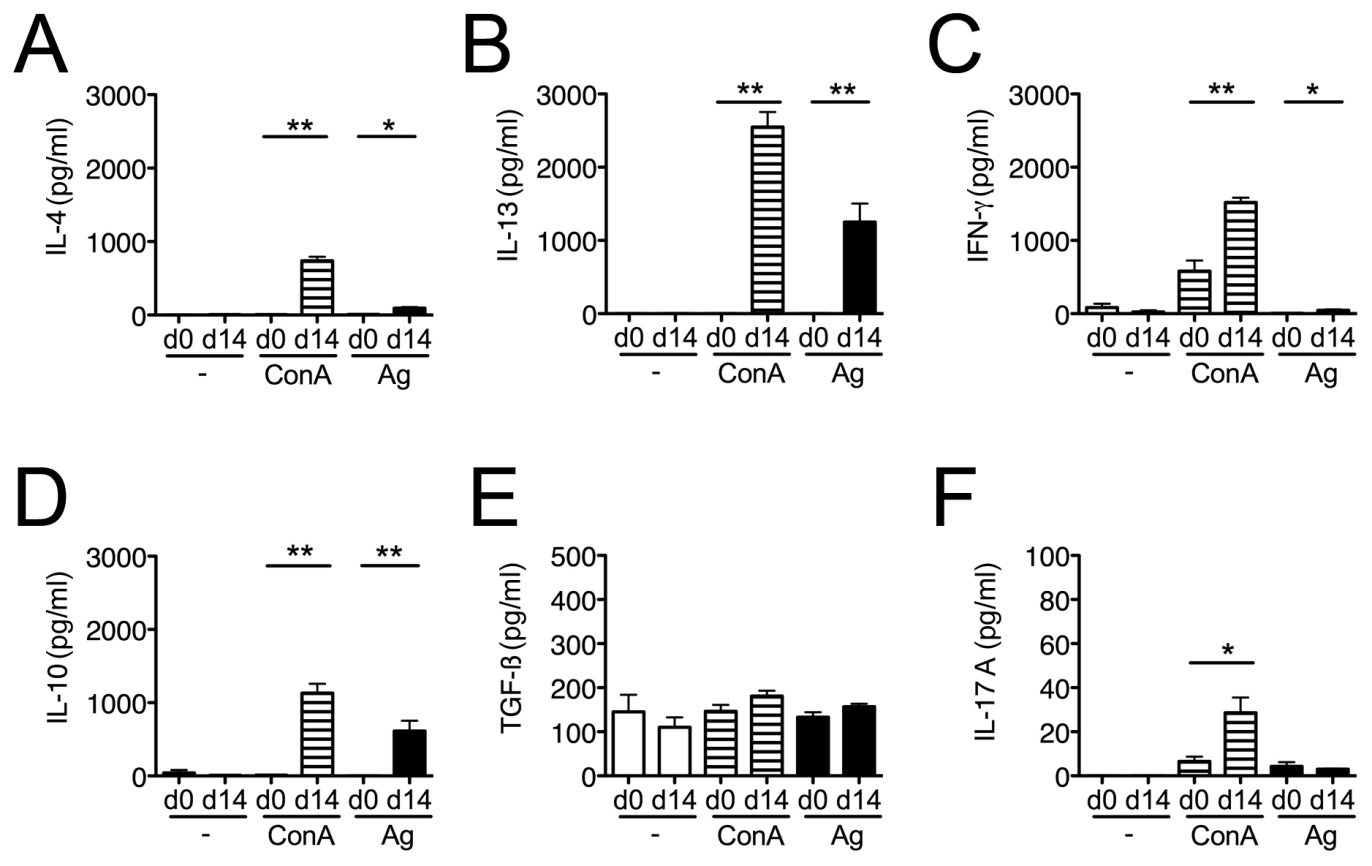
*Cytokineproduction* by mLN cells during acute *H* . *p* *. bakeri* infection. IL-4 (A), IL-13 (B), IFN-γ (C), IL-10 (D), TGF-β (E) and IL-17A (F) were measured in mLN culture supernatants with (ConA, H.p.b-Ag) or without (-) stimulation. Cells were derived from naïve controls and mice at day 14 post infection. Mean + SEM is shown for 5 mice per group. H.p.b. Ag: *H*. *p*. *bakeri* antigen; Con A: concanavalin A; * p < 0.05; ** p < 0.005.

In order to quantify the local immune response to *H* . *p* *. bakeri* we analyzed cells from the gut-draining mesenteric lymph nodes (mLN) and intestinal tissue of infected mice. On day 14 days post infection the key Th2 cytokines IL-4 and IL-13 as well as anti-inflammatory IL-10 were produced in significantly higher amounts by mLN cells from infected mice in response to mitogen and parasite antigen (Figure 2A, 2B, 2D). The levels of TGF-β did not change in acutely infected mice (Figure 2E). IFN-γ was significantly increased in mitogen-treated cultures from *H* . *p* *. bakeri* infected mice, but as expected, only negligible amounts (< 50 pg/ml) were secreted in response to parasite extracts (Figure 2C). IL-17A levels were generally very low (Figure 2 F).

Intestinal cytokine responses were analyzed in whole tissue from duodenum and cecum. Trends for increased IL-4/-13 and IL-10 production (n.s.) were detected in ileum as well as cecum tissue whereas IL-12, IFN-γ and TGF-β expression were detected at similar levels, irrespective of the infection status (data not shown).

### Cultural analysis of the intestinal microflora after nematode infection

**Figure 3:**
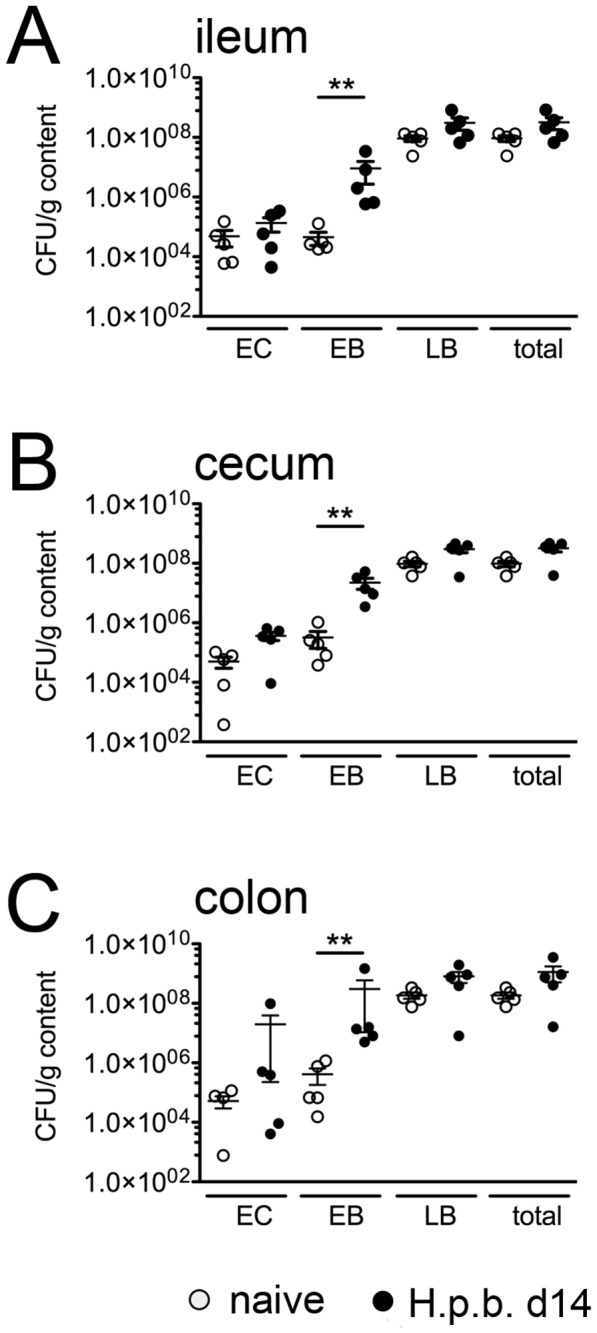
Quantification of aerobic intestinal bacterial groups. Counts of colony forming units (CFU) derived from luminal content of (A) ileum, (B) cecum and (C) colon. Open circles: naïve controls; filled circles: day 14 post *H* . *p* *. bakeri* infection. Mean ± SEM of 5 mice per group is shown. EC: Enterococci, EB: *Enterobacteriaceae*, LB: Lactobacilli; ** p < 0.005.

Given that the most pronounced small intestinal immunopathology correlated with increased levels of Th2 cytokines and IL-10 produced in mLN, we were further interested whether the acute phase of infection was accompanied by distinct shifts within the composition of the intestinal microbiota. To address this, we next quantitatively assessed the cultivable microbiota in ileal, cecal and colonic luminal contents taken on day 14 following *H* . *p* *. bakeri* infection. Infected mice displayed a trend towards higher total loads of cultivable bacteria (n.s.) as compared to uninfected controls, irrespective of the intestinal compartment analyzed (Figure 3). Strikingly, the enterobacterial loads increased significantly by 2-3 orders of magnitude in ileum, cecum as well as colon upon acute infection (Figure 3 A–C).

### Molecular analysis of the microbiota composition during *H* . *p* *. bakeri* infection

**Figure 4:**
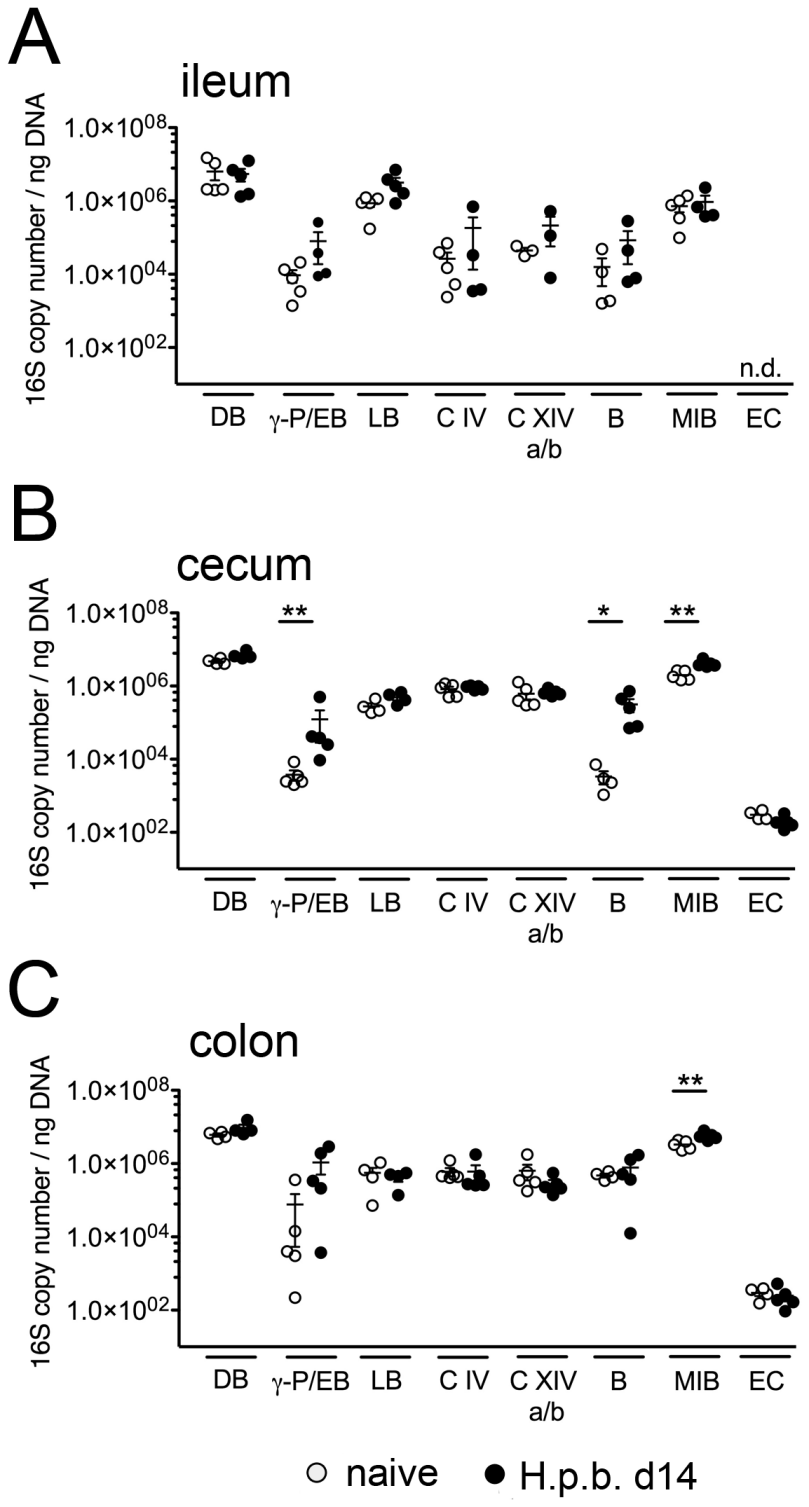
Analysis of 16S rRNA gene-based real-time PCR amplification of intestinal bacterial groups. Detected DNA levels in luminal content from (A) ileum, (B) cecum and (C) colon of naive (open circles) and acutely infected (day 14 post infection, black circles) mice were calculated as 16S rRNA copy numbers per ng DNA extract. Mean ± SEM is shown for 4-5 mice per group. DB: domain bacteria, γ-p/EB: γ*-Proteobacteria/Enterobacteriaceae*, LB: Lactobacilli, C IV: *Clostridium* cluster IV, C XIV a/b: *Clostridium* cluster XIV a/b, B: *Bacteroides* group, MIB: mouse intestinal *Bacteroides* , EC: Enterococci, n.d.: not detectable. * p < 0.05; ** p < 0.005 compared to naïve.

We next performed a comprehensive molecular survey of the microbiota composition of ileum, cecum and colon upon *H* . *p* *. bakeri* infection applying quantitative 16S rRNA technique. Irrespective of the intestinal compartment, total bacterial abundance did not change in infected mice (Figure 4). Whereas abundances of Gram-positive bacterial groups such as Lactobacilli and *Clostridium* clusters were similar in cecum and colon of naïve and *H* . *p* *. bakeri* infected mice (< 2 fold increase, Figure 4B, 4C), a trend towards higher loads was detected in the small intestine of infected mice (3.7-7.0 fold increase, n.s.; Figure 4A). Significantly increased loads of gram-negative γ*-Proteobacteria/Enterobacteriaceae* were detected in cecal contents of *H* . *p* *. bakeri* colonized animals as compared to naive mice (32.7 fold increase, Figure 4B), whereas only a trend towards higher abundance of Gram-negative enterobacterial groups upon infection could be detected in the colon (Figure 4C). In addition, the cecum of infected mice harboured significantly higher loads of the Gram-negative obligate anaerobic *Bacteroides* group (93.6 fold increase, Figure 4B) whereas members of the operational taxonomic unit mouse intestinal *Bacteroides* (MIB), had increased in the ceca and colons following infection (Figure 4B and 4C). Irrespective of the infection, Enterococci were abundant at comparable low levels in ceca and colons (Figure 4B and 4C), whereas *Bifidobacteria* could not be detected at all.

**Figure 5:**
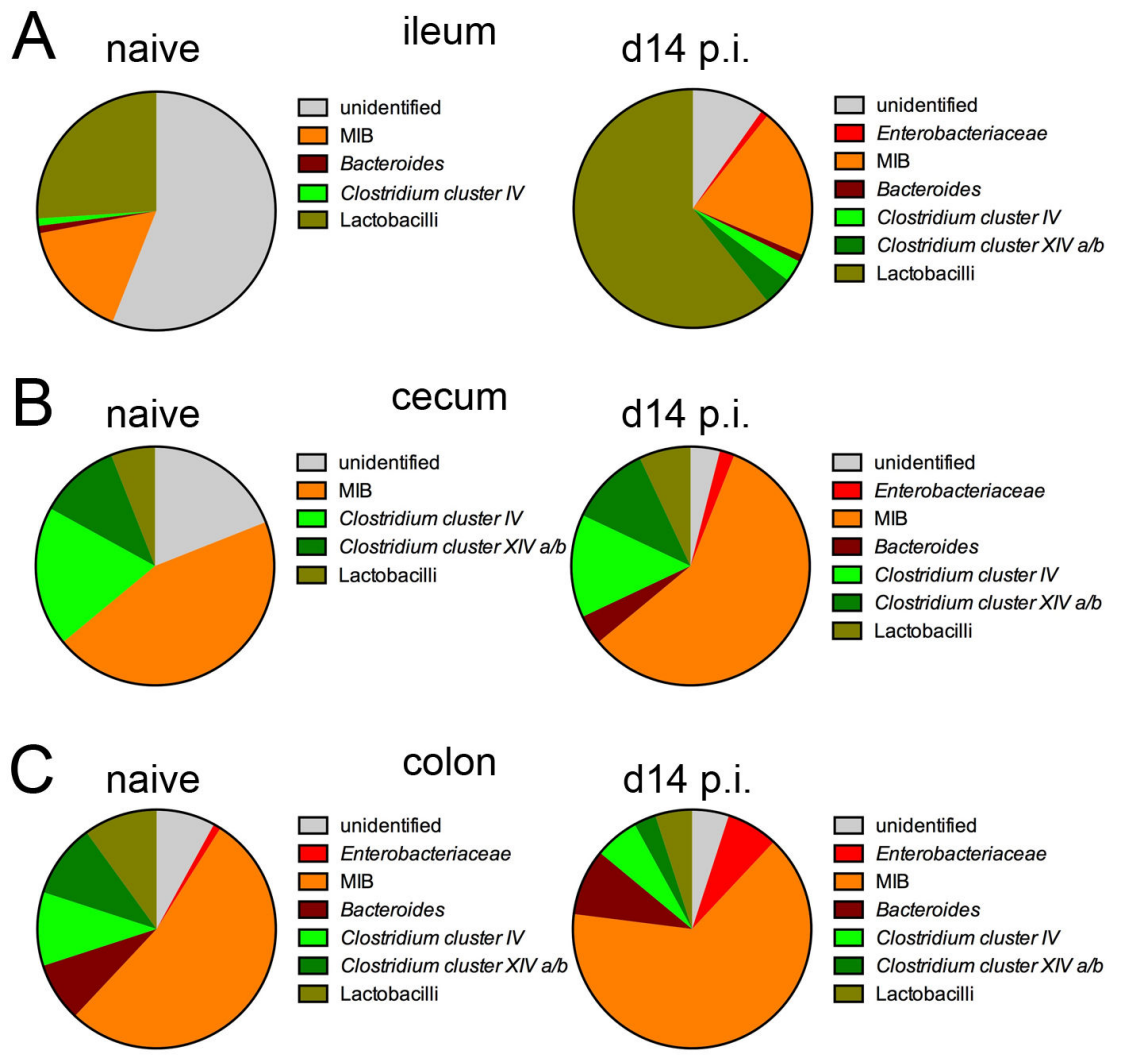
Proportions of intestinal bacterial group based on 16S rRNA gene real-time PCR. Pie charts represent the mean frequencies for the main bacterial phyla in (A) ileum, (B) cecum and (C) colon as detected in 4-5 mice per group. Abundance was calculated using the phylum-specific 16S gene copy numbers per ng DNA in relation to the amounts of the DNA copy numbers for the domain *Bacteria* set to 100%. p.i.: post *H* . *p* *. bakeri* infection.

In the following we calculated the relative abundance of the respective bacterial groups (Figure 5). The amplification with primers specific for the γ*-Proteobacteria/Enterobacteriaceae,* *Lactobacillus* group*,* *Clostridium* cluster IX*,* *Clostridium* cluster XIVa/b, MIB and *Bacteroides* group covered 81-96% of the total intestinal bacterial microbiota except for the ileal content of naïve mice, where only about 44% of the bacterial load was covered (Figure 5A). The higher coverage for ileal samples from nematode infected mice was mainly due to an increase of the *Lactobacillus* group from 26% ± 12 in naïve to 62% ± 16 in *H* . *p* *. bakeri* -infected mice (Figure 5A). Furthermore, frequencies of the cecal *Bacteroides* group were barely detectable in naïve controls but increased to about 4% in acutely infected mice (Figure 5B). Remarkably, the most distinct proportional increase for γ*-Proteobacteria/Enterobacteriaceae* was detected in the colon (from 1% ± 1 in naive to 7% ± 5 in acutely infected mice (Figure 5C)). Taken together, the data show that the applied PCR technique covered the majority of bacterial strains present in the murine intestinal tract, except for the small intestinal microbiota in naïve mice. Furthermore, the most prominent findings were increased abundances and proportions of Gram-negative γ*-Proteobacteria/Enterobacteriaceae,* *Bacteroides* and MIB in intestinal parts distal of the site of worm infection.

Given that intestinal inflammation is associated with decreased bacterial diversity [14,18,19] we finally applied denaturing gradient gel electrophoresis (DGGE), another PCR-based technique to generate genetic fingerprints of the intestinal microbiota according to the intestinal bacterial molecular band profiles before and after worm infection (Figure 1). Interestingly, six additional bands could be detected in the ileal samples derived from mice 14 day pi, which were absent in naïve controls and during the very early and chronic phase of infection (day 6 and 28 p.i., respectively) (Figure S1A). Similarly, the most distinct / overt differences were detected in cecum and colon samples at day 14 p.i. (Figure S1B, S1C). Remarkably, changes in bacterial diversity patterns of the cecal microbiota were not exclusively found during acute, but also in the early and chronic phase of infection.

DGGE bands only present in the DNA extracted from ileal contents of naïve mice could be identified as *Paenibacillus* spp. (Figure S2A), whereas additional bands present in cecal content of acutely infected mice were identified as Gram-negative *Prevotella* spp. and *Porphyromonadaceae* belonging to the *Bacteroides* group (Figure S2B). Members of *Lachnospiraceae* were detected in the cecum of all acutely infected mice, while only 2 out of 5 naïve mice showed the respective bands (Figure S2B).

### Increase in Enterobacteria after *H* . *p* *. bakeri* infection is independent of IL-4Rα^-/-^ signalling

**Figure 6:**
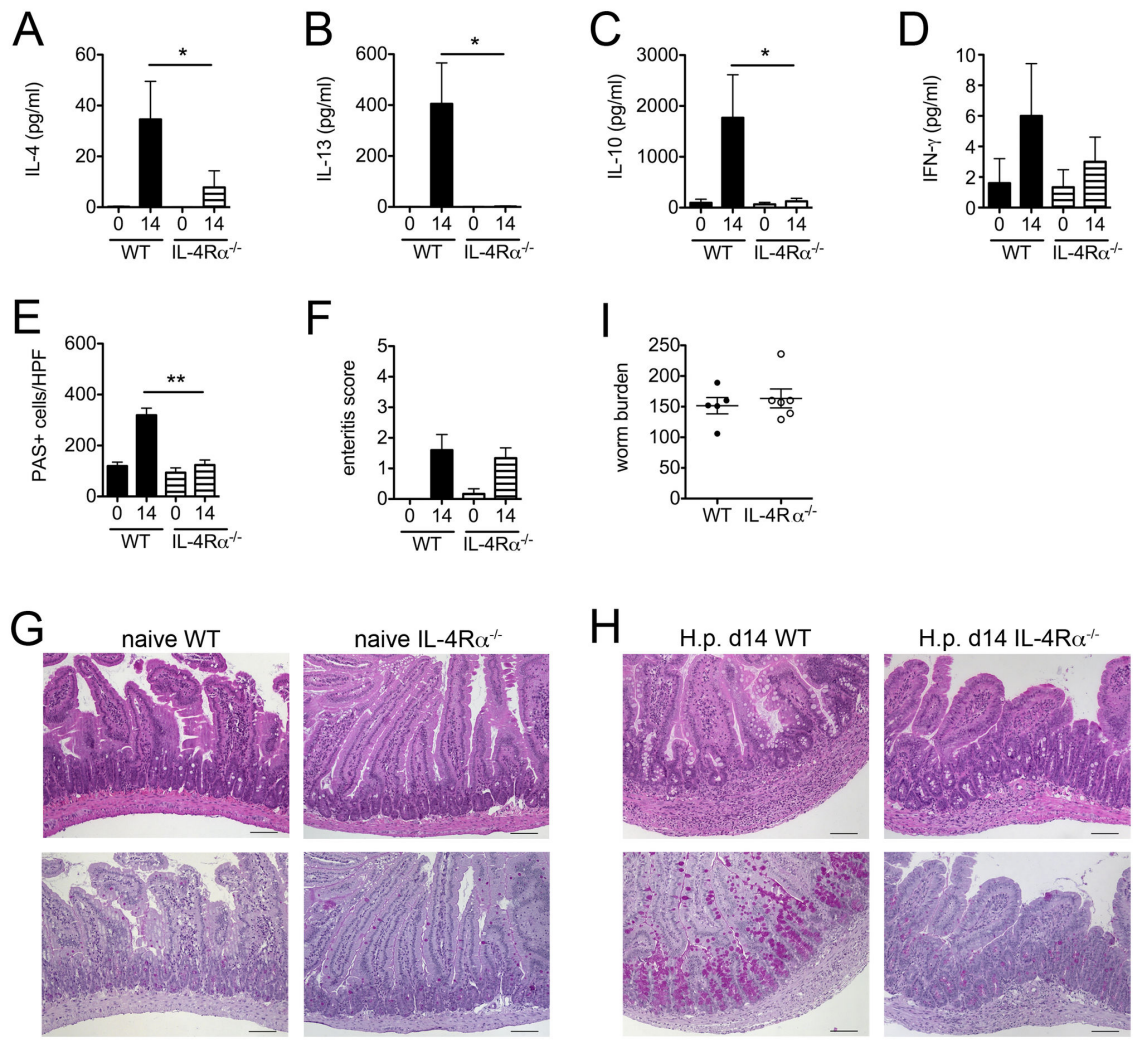
Immune response, histopathology and parasite burden of infected wild type versus IL-4Rα^-/-^ mice. Levels of IL-4 (A), IL-13 (B), IL-10 (C) and IFN-γ (D) produced by mLN cells from naïve controls and mice at day 14 post infection in response to adult worm antigen. (E) Goblet cell counts in duodenal cross sections. (F) Enteritis scores for duodenal tissue. Representative duodenum cross sections from naïve (G) and acutely infected (H) wild type and IL-4Rα^-/-^ mice stained with H&E (upper row) and periodic acid/Schiff (lower row) for histopathological scoring and goblet cell quantification. (I) Adult worm counts of WT and IL-4Rα^-/-^ mice at day 14 post infection. Mean + SEM of 5-6 mice per group is shown. * p < 0.05; ** p < 0.005.

As physiological gut functions such as mucosal permeability, chloride secretion, smooth muscle contractility and glucose absorption of the small and large intestine are altered in nematode infected mice and this is mainly mediated by IL-4/-13 [20–22], we asked whether the microbiota changes detected in the acute phase of infection with *H* . *p* *. bakeri* were depending on the IL-4/-13 – STAT6 signalling axis. Hence we analyzed the intestinal pathology and the cultivable flora of naïve and acutely infected IL-4Rα^-/-^ mice. Expectedly, IL-4Rα^-/-^ mice showed a substantially decreased Th2 cytokine response as indicaticated by significantly downreguated IL-4/-13 production of mLN cells and a diminished IL-10 response to parasite-specific restimulation as compared to wild type controls, whereas the IFN-γ levels did not differ (Figure 6 A–D). Worm burdens were comparable in wild type and IL-4Rα^-/-^ mice (Figure 6I). Interestingly, IL-4Rα^-/-^ mice displayed virtually no sign of duodenal goblet cell hyperplasia in response to *H* . *p* *. bakeri* infection (Figure 6E, 6G, 6H), but developed a locally restricted duodenal enteritis indistinguishable from the inflammatory response in infected wild type mice (Figure 6F, 6G, 6H).

**Figure 7:**
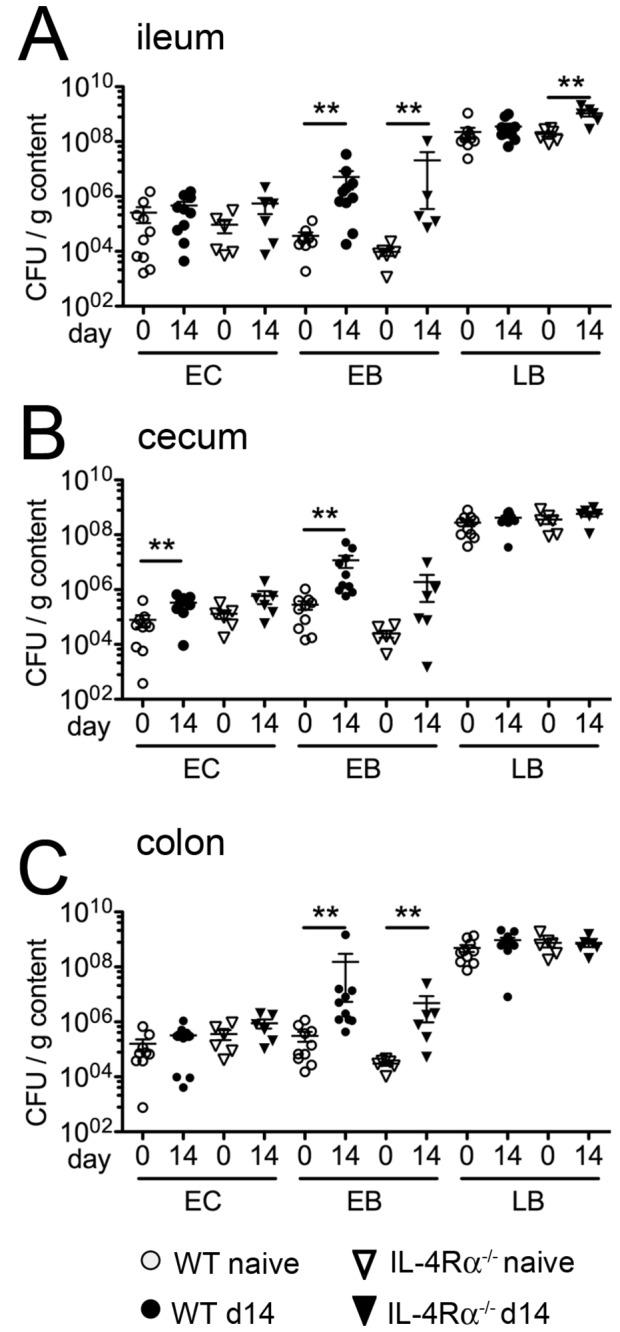
Quantification of aerobically cultivable bacteria in wild type and IL-4Rα^-/-^ mice. Counts of colony forming units (CFU) derived from luminal content of (A) ileum, (B) cecum and (C) colon of WT (naïve: open circles; d14 p.i. black circles) and IL-4Rα^-/-^ mice (naïve: open triangles; d14 p.i. black triangles). Mean ± SEM of 5-6 mice per group is shown. CFU: colony forming units, EC: enterococci, EB: *Enterobacteriaceae*, LB: Lactobacilli; ** p < 0.005.

Remarkably, the main shifts in intestinal microbiota composition observed in infected IL-4Rα^-/-^ mice were also due to increased abundances of *Enterobacteriaceae* (Figure 7) as indicated by higher enterobacterial loads in both ileum and colon, but not in the cecum (Figure 7) as compared to uninfected controls. *Lactobacilli* numbers also increased significantly in the ileal lumen of IL-4Rα^-/-^, but not wild type mice (Figure 7 A and data not shown). Taken together, the increase of intestinal *Enterobacteriaceae* after *H* . *p* *. bakeri* infection occurs independent of IL-4/-13-induced changes in the gut.

## Discussion

In the present study we analyzed whether infection with the nematode *H* . *p* *. bakeri* dwelling the proximal small intestine and the subsequently induced immune responses are associated with distinct changes of the intestinal bacterial microbiota composition. A plethora of studies in mice and men revealed that intestinal inflammation is associated with alterations in the composition of the gut microbiota towards a lower diversity and overgrowth with mainly Gram-negative species such as enterobacterial and *Bacteroides* spp. by the cost of decreasing Lactobacilli [10,14,18,19,23]. We analyzed the bacterial microbiota composition during the acute phase of the nematode infection marked by the development of a locally restricted small intestinal enteritis and the highest expression of Th2 related cytokines [17,24]. The strong Th2 response leads to changes of the small and large intestinal physiology, including increased mucosal permeability, chloride secretion and smooth muscle contractility as well as decreased glucose absorption [20–22]. We thus aimed to dissect the contribution of the highly biased Th2 immune response to alterations of the microbiota.

The identification of intestinal bacteria is impeded by the fact that most species are non-cultivable or restricted to specialized cultivation conditions. Hence, in order to analyze the complexity of microbial communities we combined classical cultivation techniques with culture-independent molecular approaches. Beside denaturing-gradient-gel-electrophoresis (DGGE) demonstrating the high diversity of the species present in the intestinal tract, a quantitative PCR technique based on the detection of 16S rRNA gene copy numbers was used for the analysis of dominant microbial groups. The latter technique quantifying γ*-Proteobacteria/Enterobacteriaceae*, the *Lactobacillus* group*,* *Clostridium* clusters IX and XIVa/b, the operational taxonomic unit MIB and the *Bacteroides* group permitted the detection of 81-96% of the total bacterial microbiota within the cecum and colon of naïve controls and acutely worm-infected mice. Of note, we restricted our analyses of infection-related changes of the microbiome to bacteria, thus possible alterations in archaeal and eukaryotic species remain to be investigated.

Intestinal inflammation may alter the total intestinal bacterial loads [12,18,19] and highly pathogenic intestinal inflammation models such as *Toxoplasma gondii*-driven ileitis and *Citrobacter* - or *Salmonella* -induced colitis are characterized by decreased loads of Gram-positive bacteria such as *Lactobacilli* and *Clostridium* groups [12,14,25]. We detected no significant effect of infections with *H* . *p* *. bakeri* on the total bacterial load in infected animals in our experiments.

In our study, the strongest alterations following acute *H* . *p* *. bakeri* infections were detected for Gram-negative bacteria. We found significant increases of cultivable *Enterobacteria* in all examined intestinal compartments, a finding confirmed by quantitative PCR for γ*-Proteobacteria/Enterobacteriaceae*. IBD-like inflammation models, regardless whether induced by infections, chemicals or genetic predisposition [10,14,19], as well as human IBD [26–28] are characterized by overgrowth of Gram-negative *Enterobacteriaceae* and such changes in the intestinal microbiota may be associated with the induction or perpetuation of the inflammatory response in IBD. It thus is interesting that a nematode infection leading to a mild and locally restricted inflammatory response also led to a significant increase in bacteria associated with inflammatory disorders like IBD. Of note, infections with *H* . *p* *. bakeri* have been shown to suppress inflammation in several models of colitis (reviewed in [9]) which are associated with increased intestinal loads of Gram-negative *Enterobacteriaceae* [10,29]. Future work will have to elucidate whether the nematode infection also affects the microbiota composition in models of colitis.

A study by Walk and colleagues previously described alterations in the gut microbiota after infection with *H* . *p* *. bakeri* [30]. In this study, a significantly increased abundance of members of the *Lactobacillaceae* family was detected with a 16s rRNA clone library generated from terminal ileum tissue samples from acutely worm infected mice after removal of the luminal content. Our data based on the analysis of luminal content also show an increase in the relative distribution of *Lactobacillaceae* members in the ileum. Sequencing of DGGE-derived DNA-bands detectable predominantly or exclusively in acutely nematode-infected mice revealed an increase in *Lachnospiraceae* and *Porphyromonaceae* members after infection, which confirms the data published by Walk et al. [30]. Differing compositions of the gut-wall-associated versus luminal flora and the relatively low abundance of *Enterobacteriaceae* in the gut of naïve and infected mice may explain why we detected changes concerning this bacterial group that have not been detected in the previously published study.

It is up to speculation whether inflammation-driven changes in the gut physiology after *H* . *p* *. bakeri* infection directly facilitate the outgrowth of Gram-negative bacteria or act indirectly by creating conditions less well tolerated by other groups of commensals. *Enterobacteriaceae* as facultative anaerobes use mixed-acid fermentation under anaerobic conditions to produce lactate, succinate, ethanol, acetate and carbon dioxide. These pathways require low levels of hydrogen. One factor facilitating the outgrowth of *Enterobacteriaceae* in the inflamed gut may be that the members of this group are relatively resistant to oxidative stress mediated by reactive oxygen metabolites, which are produced during inflammatory processes [31,32]. Another reason might be their high efficiency in metabolizing glucose, which is absorbed to a lesser extent during intestinal inflammation [33]. Studies in pigs infected with the nematode *Trichuris* *suis* demonstrated changes in both microbial composition and metabolic potential in the lumen of the colon [34,35]. Members of the *Enterobacteriaceae*, such as *E. coli*, might profit from an increased fluid influx and impeded glucose absorption induced by infection with *H* . *p* *. bakeri* [20]. These effects, together with an increased mucus production and gut peristaltic, largely depend on the key Th2 cytokines IL-4/-13 and STAT-6 signalling. However, we found no evidence for a role of IL-4/-13 in the detected microbiota changes, as IL-4Rα^-/-^ mice showed similar alterations after infection with *H* . *p* *. bakeri* .

Infections with another intestinal nematode, *T* *. muris* , alter the expression of anti-microbial products such as angiogenin 4 and cryptdins in the intestine [36,37]. We hence asked whether the changes in the microbiota composition after infection with *H* . *p* *. bakeri* correlated with altered expression of anti-microbial defence molecules and focussed on members of the cryptdins, angiogenins and cathelecidins with known activity against Gram-negative bacteria such as *E. coli*, *Citrobacter rodentium* and members of the *Bacteroides* family [38–40]. Analysing the cryptin DefB1, ang4 and CRAMP produced by Paneth cells and macrophages/neutrophils, respectively, we found an unaltered expression in worm-infected mice compared to naïve controls (data not shown). Thus it is unlikely that changes in the expression of innate defence molecules are crucially involved in permitting the detected changes in the composition of the intestinal bacterial community.

We and others reported on *H* . *p* *. bakeri* -driven modulations of the host immune response during concurrent intestinal infections [41–43]. On the one hand *H* . *p* *. bakeri* may impair host resistance to bacterial pathogens, such as *Citrobacter rodentium* and thereby enhance *Citrobacter* -induced colitis [42] with altered macrophages being incapable to kill internalized pathogenic bacteria. On the other hand, the nematode protects the host from *Helicobacter pylori*-driven inflammation [43]. Furthermore, *H* . *p* *. bakeri* effectively suppresses intestinal inflammation in murine models of human IBD [44–47]. The fact that *H* . *p* *. bakeri* infection leads to the atypical production of immunosuppressive TGF-β by intestinal CD4^+^ T cells in response to TLR-4 triggering by LPS [48] poses the question whether such mechanisms of immune modulation help to avoid undesired immune activation by microbiota changes resulting from the infection. It remains to be investigated whether nematodes also interfere with the composition of the intestinal microbiota in face of a dysregulated intestinal immune response. Evidence comes from a study showing that *Trichuris* *trichiura* infections in macaques suffering from chronic diarrhea alters the composition of the bacterial microbiota attached to the mucosa, which correlated with a clinical improvement of chronic diarrhea [49].

In conclusion, our study analyzing the ileal, cecal and colonic microbiota composition during an *H* . *p* *. bakeri* infection permits the following insights into the parasite-bacteria interplay: I) An intestinal nematode infection leads to moderate changes in the complexity of the intestinal microbiota and no significant alterations in the total number of intestinal bacteria. II) The peak of pathology induced by the intestinal nematode infection is accompanied by a significant increase in gram-negative bacteria including γ*-Proteobacteria/Enterobacteriaceae*. III) Changes in microbiota composition occur not only in the proximity of the nematode’s habitat, but also in distal regions of the gut. VI) The detected microbiota alterations in nematode infected mice are not a consequence of IL-4/-13-depending changes in the intestine. The impact of nematode-induced microbiota changes on IBD remains to be investigated, possibly adding to our understanding of how theses parasites interfere with intestinal inflammation.

## Supporting Information

Figure S1 **DGGE analysis of luminal contents of (A) ileum, (B) cecum and (C) colon.** Band profiles shown derive from 3 naïve and infected mice at different time points after infection. Arrowheads mark additional bands during acute *H* . *p* *. bakeri* infection (14 d.p.i.). M: marker.
(TIF)

Figure S2 **DGGE profiles and results of sequencing analysis of naive versus acutely infected mice.** SYBR green stained gels of extracted DNA from (A) ileum and (B) cecum were used for DNA extraction and sequencing of the marked bands.
(TIF)
