# Supplementary figures and images for: Small Intestinal Nematode Infection of Mice Is Associated with Increased Enterobacterial Loads alongside the Intestinal Tract
Source: PLoS One. 2013 Sep 10;8(9):e74026. doi: 10.1371/journal.pone.0074026 (PMC3769368; doi:10.1371/journal.pone.0074026)

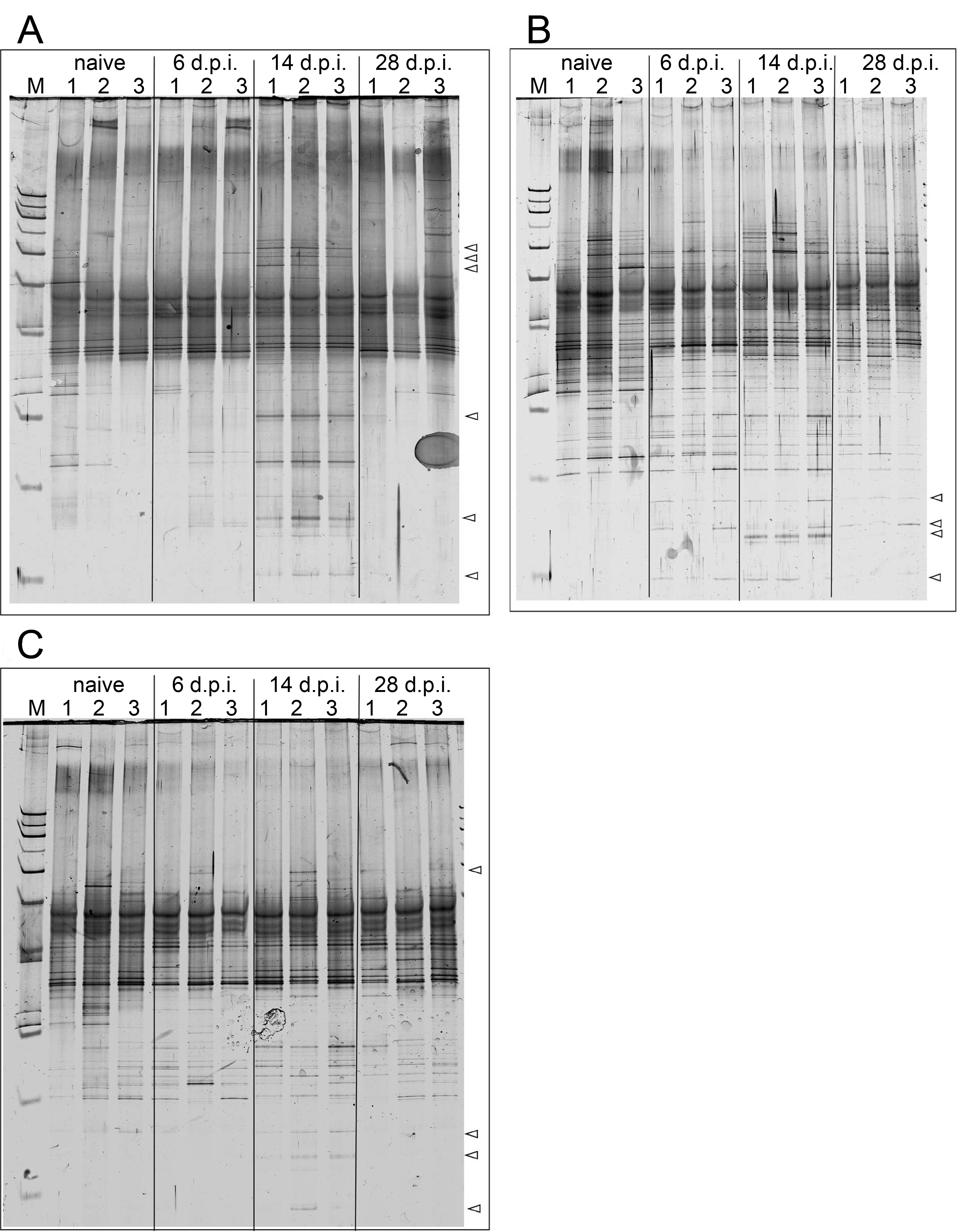

Supplement: Figure S1 — DGGE analysis of luminal contents of (A) ileum, (B) cecum and (C) colon. Band profiles shown derive from 3 naïve and infected mice at different time points after infection. Arrowheads mark additional bands during acute H . p . bakeri infection (14 d.p.i.). M: marker. (TIF) [file pone.0074026.s001.tif]

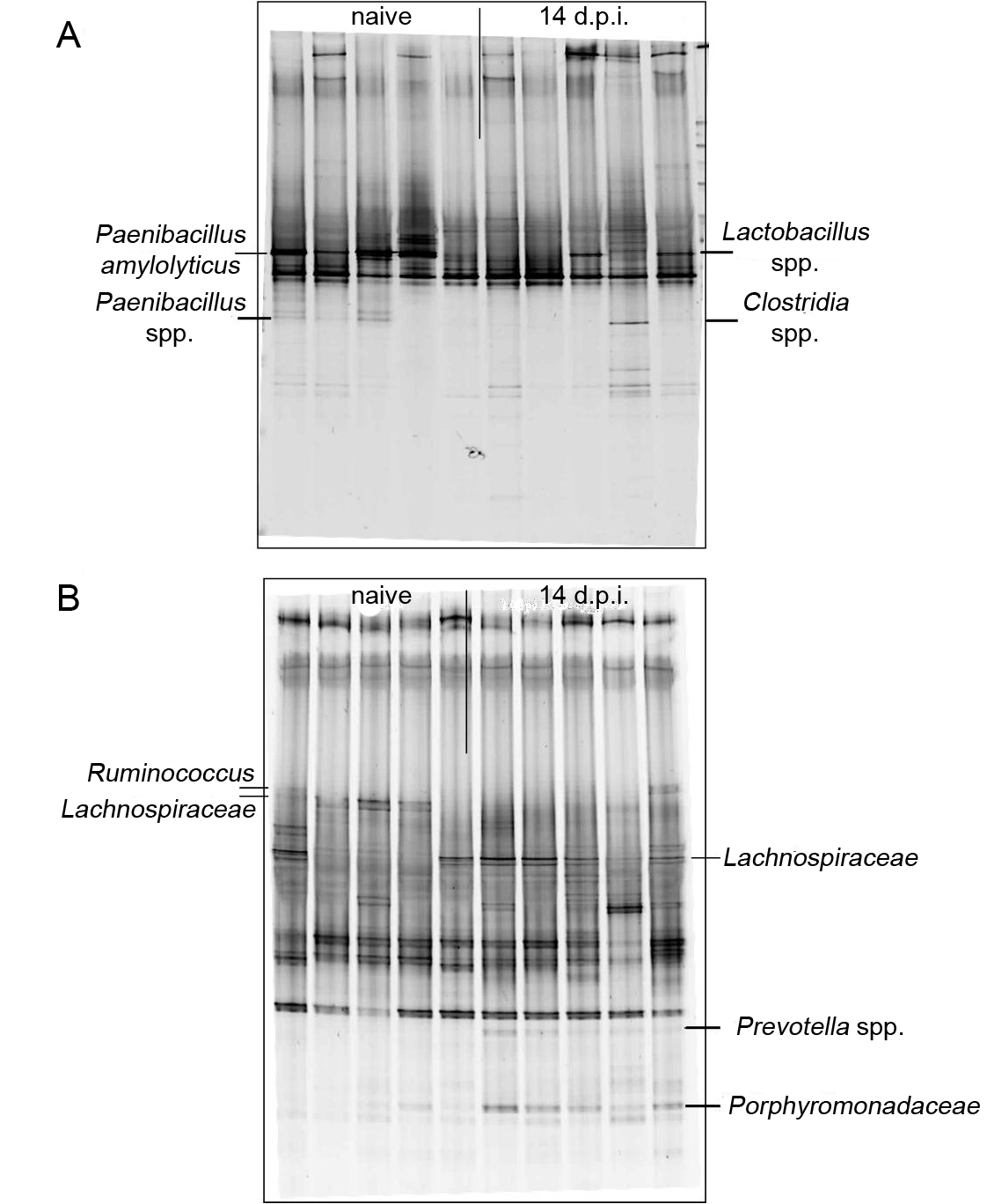

Supplement: Figure S2 — DGGE profiles and results of sequencing analysis of naive versus acutely infected mice. SYBR green stained gels of extracted DNA from (A) ileum and (B) cecum were used for DNA extraction and sequencing of the marked bands. (TIF) [file pone.0074026.s002.tif]
